# Anger and Aggression in Relation to Psychological Resilience and Alcohol Abuse among Health Professionals during the First Pandemic Wave

**DOI:** 10.3390/healthcare11142031

**Published:** 2023-07-15

**Authors:** Argyro Pachi, Evgenia Kavourgia, Dionisios Bratis, Konstantinos Fytsilis, Styliani Maria Papageorgiou, Dimitra Lekka, Christos Sikaras, Athanasios Tselebis

**Affiliations:** 1 Psychiatric Department, Sotiria Thoracic Diseases Hospital of Athens, 11527 Athens, Greece; irapah67@otenet.gr (A.P.); ekavourgia@gmail.com (E.K.); dionbratis@yahoo.gr (D.B.); kosfits@gmail.com (K.F.); stellamar4@yahoo.gr (S.M.P.); lekkadim@yahoo.gr (D.L.); 2 Nursing Department, Sotiria Thoracic Diseases Hospital of Athens, 11527 Athens, Greece; cris.sikaras@gmail.com

**Keywords:** psychological resilience, anger, aggression, alcohol abuse

## Abstract

Mental health problems, behavior changes, and addictive issues have been consistently documented among healthcare workers during the pandemic. The objective of this study was to investigate the levels of anger and aggression in relation to psychological resilience and alcohol abuse among healthcare workers during the first wave of the COVID-19 pandemic. A total of 120 physicians and 123 nurses completed an online survey of the Dimensions of Anger Reactions-5 (DAR-5), the Brief Aggression Questionnaire (BAQ), the Brief Resilience Scale (BRS), and the Alcohol Screening questionnaire CAGE which is an acronym for the focus of the questions (Cutting down, Annoyance by criticism, Guilty feeling, and Eye-openers). Demographic and professional data were also recorded. A total of 53 men and 190 women participated in the study. Almost one-third of the participants had a positive score on the DAR-5 scale and one out of ten respondents presented with current problematic alcohol use. Male participants demonstrated lower scores on the DAR-5 scale compared to females. Individuals with current problematic alcohol use displayed higher scores on the BAQ compared to those without alcohol use disorders. Regression analysis revealed that 16.4% of the variance in the BAQ scores can be attributed to scores on the DAR-5, 5.9% to the BRS scores, 2.1% to the CAGE scores, 1.7% to gender, and 1.2% to years of work experience. Mediation analysis highlighted the role of psychological resilience as a negative mediator in the DAR-5 and BAQ relationship. Professional experience and alcohol abuse emerged as positive and negative risk factors contributing to aggression and psychological resilience. The findings hold practical implications for implementing interventions to strengthen resilience in order to compensate for aggressive tendencies and discourage addictive issues.

## 1. Introduction

The COVID-19 pandemic and other previous epidemics have primarily and disproportionately exerted their deleterious effects upon healthcare professionals, who are the first line of defense committed to handling the health emergency situation [1]. Meanwhile, recently available studies identified a high prevalence of insomnia, stress, anxiety, depression, and trauma, along with other related psychological health problems, health behavior changes, and addictive issues among healthcare workers [2,3,4,5]. During the pandemic research evidenced that anxiety and depression contributed to increased alcohol consumption and heavy alcohol use exacerbated depression, anxiety, and insomnia, constituting a bidirectional association [6,7,8,9]. 

Literature suggests that exposure to stress, especially from fatal, catastrophic events, elevates the risk of subsequent alcohol consumption and alcohol use disorders [10,11,12]. During the pandemic, health professionals experienced an immense amount of stress either directly related to the nature of their work or applicable to every human being in the general population [13,14,15,16]. Under conditions of increased job strains, when employees are confronted with complicated, controversial, and equivocal situations, they may experience work stress [17]. Sources of occupational stress in healthcare personnel during the pandemic, especially in the first wave, include fear of infection and its transmission, lack of protective equipment and its use during working hours, frequent changes in health protocols, inability to provide the standard level of care due to limited material and human resources availability, visiting hours for the critically ill patients, increased workload, moral injury, social exclusion, and social isolation measures [18]. 

In the meantime, symptoms of anger and confrontation have been reported in more than half of participants in a national cross-sectional survey in the United Kingdom during the first pandemic wave [19]. Anger, either state or trait, is regarded as a social emotion, predisposing to aggressive behavior. It may occur as a result of being subjected to a real or perceived threat, but it may also be a sign of a mental health disorder such as anxiety or depression. Earlier research findings supported the idea that anger, cynicism, and emotional exhaustion among hospital staff predicted distress levels resulting from increased workload and these negative distress reactions contributed substantially to increased levels of depression [20]. Anger as a consequence of perceived health threat has been documented, especially among nurses who were quarantined, in previous outbreaks of Severe Acute Respiratory Syndrome [21,22]. Recent research, during the COVID-19 pandemic, investigated the impact of psychological fatigue on the association between work stress, anger, and voluntary job attrition among nursing staff [23]. Factors like occupational stress and anger contribute to emotional exhaustion and moreover, anger may evoke adverse interactions among employees, motivating staff to exhibit aggressive behaviors [24,25,26]. Since ethical concerns are raised when healthcare personnel express anger, the relevant scientific literature is limited [27]. Yet, there is evidence that healthcare workers can experience angry emotions because of the stressful working conditions and during the pandemic such uncomfortable emotional experiences appear to arise [28,29,30]. In a recent study from the early phase of the pandemic, 15% among 14,600 healthcare workers admitted experiencing anger the day before completing the survey [31]. 

Hopefully, feelings of anger do not necessarily result in aggressive behavior. Aggression as a concept refers to a pattern of hostile, harmful, or destructive behaviors, usually meant to inflict deleterious repercussions. Recent aggression models, such as the General Aggression Model, argue that aggression is derived from combined biological, situational, and individual factors, in particular personal traits, prior life experiences and environmental inputs [32]. Workplace aggression against healthcare professionals is a well-known and ever-present problem, on the rise during the pandemic, threatening wellbeing and the right to work in a safe environment [33,34,35,36]. Plenty of research data refer to type II aggression, from patients or relatives against health staff [37]. Yet, information on aggression from healthcare professionals directed towards others and evidence based on type III aggression, particularly with reference to the COVID-19 pandemic, is limited [38,39,40,41,42,43,44,45]. 

Studies conducted among healthcare workers, after the onset of the COVID-19 pandemic, evidenced an escalation in alcohol consumption, presumably as a result of maladaptive attempts to relieve stress and overcome the increased psychological difficulties resulting from the pandemic [46,47,48]. Previous research advanced the role of anxiety symptoms, in contributing to problem drinking and alcohol-related aggression [49]. Alcohol use is more strongly linked to aggressive behavior than any other psychoactive agent, although the manifestation of aggression depends on individuals and circumstances [50,51,52]. Studies support that alcohol consumption elevates the risk for aggressive behavior, especially in men with increased dispositional anger, poor anger control, low consideration for future consequences, low psychological flexibility, and impaired executive functioning resulting in disinhibition and impulsivity [53,54,55,56,57]. Furthermore, it has been suggested that individuals with a genetic vulnerability to stressful situations due to their hypofunctional serotonergic neurotransmission linked with decreased alcohol-induced sedation, when facing negative life events have the propensity to consume excessive amounts of alcohol and exhibit aggressive behavior at the same time [58].

Psychological resilience is expected to provide protection in traumatic and distressing situations and earlier research supported that resilience has been proven to effectively protect healthcare staff from psychological distress, anxiety, depression, and burnout [59,60]. During the pandemic, research studies argue that healthcare workers with high levels of resilience have an advantage over their low-resilient counterparts to successfully rebound and withstand the pandemic psychological strain, but since psychological resilience is a dynamic construct, the reverse paradigm also applies, meaning that certain factors may weaken resilience and influence coping strategies [61,62,63]. 

The effect of resilience in the relation between occupational stress and anger in first responder populations in emergency situations has already been studied and as suggested in previous research resilience to challenging workplace situations is negatively related with state and trait anger [64,65]. Also, during the pandemic, studies support that psychological resilience has the ability to attenuate feelings of anger and counteract emotions of fear and worry caused by the pandemic preventing the emergence of stress, anxiety, and depression [66,67,68,69]. Likewise, psychoeducational interventions such as anger control training programs reduced the amount of anger and increased the level of resilience [70]. 

Studies confirm significant negative associations between aggression and psychological resilience and argue that spirituality and self-control may have a positive impact on reducing aggression [71,72,73]. From a different perspective, another way of reducing aggression in healthcare professionals, working during the COVID-19 pandemic, is the enhancement of their level of resilience to counteract aggression [74,75]. In order to reinforce psychological resilience improving sleep quality and following the directives of positive psychology are a few among other health promotion interventions [76,77,78]. Furthermore, previous and recent studies during the pandemic evidence that higher levels of psychological resilience buffer against stress and negative emotions and have been associated with lower alcohol consumption and lower hazardous drinking patterns [79,80,81,82,83,84,85]. 

The conceptual framework for our study was that enhanced psychological resilience may effectively mitigate anger and counteract aggression, even in adverse circumstances, but the question is if its protective role is compromised by certain risk factors such as alcohol abuse. The above hypothetical question underlies the main purpose of the study which is to have the opportunity to concurrently investigate the protective role of psychological resilience and the impact of alcohol abuse on aggression and their possible emerging interactions. Secondary objectives of the present study are to:

1. Evaluate the levels of anger, aggression, psychological resilience, and alcohol abuse among healthcare professionals during the first wave of the COVID-19 pandemic. 

2. Explore for other demographic and work-related factors potentially contributing to aggression and psychological resilience. 

The scientific significance of the study becomes apparent considering the limited research on healthcare workers’ aggression towards others in conjunction with the investigation of potentially modifiable risk and protective factors such as alcohol abuse and psychological resilience. 

## 2. Subjects and Methods

### 2.1. Research Design

This was a cross-sectional correlational design study, conducted from 15 June to 30 June 2020, through web-based self-report surveys administered via email. The authors designed the survey draft, and its final version was approved by consensus. The email addresses of the participants were retrieved through links to the websites of Greek healthcare workers from their scientific and professional associations. The first page of the electronic survey provided information about its scientific purpose and its voluntary nature. Also, the first page of the electronic questionnaire included a consent form to participate where we ensured that participants gave online informed consent by responding to the question: “Do you agree to participate in this study?” which was the first question in the online questionnaire. Participants with positive responses could then participate in our study. The study sample consisted of medical and nursing staff who agreed to respond to the email as a convenience sample and no measures were taken to increase the response rate, apart from the reassurance of data privacy. Τhe invitation was sent as an electronic message also contained an anonymous link allowing access to the online survey platform. 

The study protocol has been approved by the Clinical Research Ethics Committee of the “Sotiria” General Hospital (Number 12253/7-5-20). This study was conducted in accordance with the ethical principles as defined by the Declaration of Helsinki, the International Committee of Medical Journal Editors, and the General Data Protection Regulation (GDPR-2016/679) of the European Union.

The study was conducted during the gradual de-escalation of the restrictive measures implemented by the Greek authorities. The lockdown in Greece was imposed from 23 March until 4 May 2020 and was characterized as one of the most restrictive in Europe, attempting to control the infection and the mortality rate. In a pilot study of ours [86], during the lockdown in April 2020, we investigated the relationship between anger, aggression, resilience, and family support in a general and healthcare population sample and the results reported higher anger scores among healthcare workers compared to the general population, which motivated us to study these and other related variables exclusively among healthcare workers. 

### 2.2. Study Participants

A total of 150 doctors and 250 nurses were invited to participate in the study, 243 of whom (120 doctors and 123 nurses) responded to the invitation. Sample adequacy was calculated using the G-Power Version 3.1 software [87]. With a sample of 243 subjects, seven factors, and an alpha of 0.05, and the calculated power was 1.00. A Monte Carlo power analysis was performed for a single-mediated model [88]. For a sample of 243 subjects, 5000 of replications and a 95% confidence level the calculated power was 0.93.

### 2.3. Measurement Tools

Participants were invited, before completing the questionnaires, to state their demographic and professional data including age, gender, professional status and working experience in years.

#### 2.3.1. Dimensions of Anger Reactions-5 (DAR-5)

The Dimensions of Anger Reactions-5 (DAR-5) is a 5-item self-administered scale designed to measure the experience of anger over the past four weeks, by answering on a 5-point Likert scale grading from 1 (None or almost none of the time) to 5 (All or almost all of the time). Total scores vary from 5 to 25, with higher scores reflecting more anger experiences. A cut-off value of 12 classifies respondents as high or low scorers [89]. The DAR-5 has demonstrated sound psychometric properties and has also been evaluated across community samples and trauma-exposed populations [90,91]. Cronbach’s alpha in this study was 0.762.

#### 2.3.2. Brief Aggression Questionnaire

The Brief Aggression Questionnaire (BAQ) is a 12 item self-report measure of trait aggression. Participants are prompt to rate on a scale from 1 (strongly agree) to 5 (strongly disagree), the extent to which statements describing behaviors and emotions, are characteristic of themselves. The BAQ measures aggression on the dimensions of physical aggression, verbal aggression, anger, and hostility. The BAQ has been postulated as a valid and reliable tool, with good test–retest reliability and convergent validity with other behavioral measures of aggression [92,93,94]. Cronbach’s alpha in this study was 0.761.

#### 2.3.3. Brief Resilience Scale (BRS)

The Brief Resilience Scale (BRS) is a 6-item self-report measure of resilience, reflecting the competence to overcome stress and adversity. Responses are rated on a 5-point Likert scale from 1 (Strongly Disagree) to 5 (Strongly Agree), but half of the items are reversed scored. Scores range from 6–30, with higher scores suggesting a higher level of resilience. The total score needs to be divided by the total number of questions answered and scores between 1.00 and 2.99 represent low resilience, between 3.00 and 4.30 normal resilience and between 4.31 and 5.00 high resilience. The original BRS demonstrated good levels of internal consistency and test–retest reliability, and adequate factorial, convergent and discriminant validity [95]. BRS was translated into Greek by Stalikas and Kyriazos [96]. Cronbach’s alpha in this study was 0.856. 

#### 2.3.4. CAGE Questionnaire

The CAGE questionnaire is a brief and popular screening instrument for detecting alcohol abuse and dependence that is easily applied in clinical practice [97]. It consists of four dichotomous questions that can be answered with yes/no. Scores range from 0 to 4 with higher scores indicating more pronounced alcohol use disorders. A cutoff ≥ 2 is considered clinically significant and is recommended to detect alcohol abuse or dependence in order to provide the best combination of sensitivity, specificity, and positive predictive value [98,99]. The Kuder–Richarson coefficient of reliability (KR-20) in this study was 0.63.

### 2.4. Statistical Analysis

All variables in the analysis were computed with descriptive statistics. To enable statistical analysis of the data and to make comparisons easier, some independent variables were grouped. The variable alcohol abuse/dependence was recoded as two categories for the associations: low-risk alcohol abuse when scores were below two on the CAGE questionnaire and risky alcohol abuse when scores were above or equal to two. Likewise, the resilience variable was also recoded into three categories, for the same purpose: low, moderate, and high levels of resilience. The Fisher’s exact test was utilized to establish the differences between qualitative data. In order to assess differences between variables, Student’s *t*-test and analysis of variance (ANOVA) were employed. The assumption testing for equality of error variances using Levene’s Test was performed before the analyses. Independent sample *t*-tests assessed for differences in gender and differences between healthcare workers as to their profession and alcohol use. The effect size was calculated with Hedges’ g-value from the results of the independent samples *t*-test, considering that values less than 0.2 suggest a small effect size, less than 0.5 a medium effect size, and less than 0.8 a large effect size. The results of the ANOVA test were estimated with the eta squared value (ηp^2^), counting ηp^2^ = 0.01 as a small effect size, ηp^2^ = 0.06 as a medium effect size, and ηp^2^ ≥ 0.14 as a large effect size. To investigate if there is an interaction effect between the two independent variables (levels of psychological resilience and alcohol abuse) in terms of a continuous dependent variable (aggression), whilst adjusting for continuous covariates that are thought to influence this interaction effect, a two-way analysis of covariance (ANCOVA) was employed. The assumption testing for a two-way ANCOVA (linearity, homogeneity of regression slopes, homogeneity of variances using Levene’s test of equality of variances, homoscedasticity, absence of significant outliers, and normal distribution of residuals) was carried out before the analysis. Pearson correlation was conducted to assess the direction and strength of the relationship between variables and the values of the correlation coefficients were interpreted as weak from ±0.10 to ±0.29, moderate from ±0.30 to ±0.49, and strong from ±0.50 to ±1.00. Linear regression models were built to explore if the related variables were significant predictors of the dependent variable, aggression. The assumption testing for regression analysis (linear relationship, independence, homoscedasticity, and normality) was carried out performing a graphical review of the variables, residuals and collinearity statistics, probability–probability (PP) plots, and scatterplots. A simple mediation analysis using the Hayes SPSS Process Macro was performed to investigate the role of resilience in the relationship between anger and aggression, setting anger as the predictor variable, aggression as the outcome variable and resilience as the mediator variable for the analysis. Statistical significance level was set at *p* < 0.05 and *p* values were two-tailed. Analyses were performed using IBM SPSS Statistics 23 (IBM SPSS Statistics for Windows, Version 23.0). Mediation analysis was conducted using the Hayes SPSS Process Macro [100]. IBM SPSS AMOS 23 Graphics enabled the graphical representation of the mediation analysis.

## 3. Results

### 3.1. General Characteristics of Participants and Scores on Outcome Variables as to Gender and Alcohol Abuse 

The study included a total of 243 participants (53 men and 190 women). As to the profession, 120 of them belonged to the medical and 123 to the nursing staff. Means and standard deviations for general characteristics of participants and all outcome variables as to gender are presented in Table 1. A positive score value on the DAR-5 scale was reported by 32.5% of participants. A total of 9.9% of responders scored above cut-off on the CAGE questionnaire. A total of 15.4% of current alcohol users felt the need to cut down. 9.7% of them have been annoyed by people criticizing their drinking, 12.6% have felt bad or guilty about their drinking, and a few of them (1.6%) had the need for alcohol in the morning as an eye opener. Means and standard deviations for general characteristics of participants and all outcome variables as to alcohol abuse are presented in Table 2. A total of 18.1% of respondents were classified as low resilient individuals (11.32% of male participants versus 20% of females, *p* = 0.164), 58.4% as normal (56.6% of males as to 59% of females, *p* = 0.756), and 23.5% as high resilient (32.07% of males compared to 21% of females, *p* = 0.102), concluding that gender differences in levels of resilience did not reach statistical significance.

Female participants evidenced higher scores on the DAR-5 scale compared with their male counterparts (10.37 ± 3.50 versus 9.38 ± 2.52, *t*-test *p* < 0.05, Hedges’ g: 0.30), (Table 1). Also, female healthcare workers displayed higher scores on the hostility subscale of the BAQ scale compared with males (6.72 ± 2.6 versus 5.72 ± 2.14, *p* < 0.01, Hedges’ g: 0.43), (Table 1), whereas males scored higher on the physical aggression subscale of the BAQ scale compared with females (5.45 ± 2.47 versus 4.11 ± 1.87, *p* < 0.01, Hedges’ g: 0.66), (Table 1). Levene’s test showed that the variances were equal (*p* > 0.05).

Healthcare workers with alcohol use disorders scored higher on the BAQ scale and its physical aggression subscale compared to participants without alcohol abuse (27.5 ± 7.95 versus 23.11 ± 6.69, *p* < 0.05, Hedges’ g: 0.66 and 5.83 ± 2.74 versus 4.24 ± 1.95, *p* < 0.01, Hedges’ g: 0.78), (Table 2). As to participants’ professions, no differences were observed in key outcome variables, except for the fact that nursing staff participants were older and had more years of professional experience compared to physicians (*t*-test *p* < 0.01). Levene’s test showed that the variances were equal (*p* > 0.05).

### 3.2. Differences on Outcome Variables as to the Level of Resilience

Meanwhile, participants’ level of resilience revealed significant differences, as determined by one-way ANOVA, the DAR-5 scores (*F*_(2, 240)_ = 5.296, *p* < 0.01, ηp^2^= 0.042), the BAQ scores (*F*_(2, 240)_ = 4.892, *p* < 0.001, ηp^2^ = 0.068), and on working experience (*F*_(2, 238)_ = 5.296, *p* = 0.012, ηp^2^ = 0.036). A Tukey post hoc test identified statistically significant differences among normal (9.92 ± 3.332, *p* < 0.01) and high resilient groups (9.65 ± 2.9, *p* = 0.01), compared to the low resilience group (11.59 ± 3.526) as to the DAR-5 scores, among normal (22.85 ± 6.527, *p* < 0.001) and high resilience groups (22.33 ± 6.61, *p* = 0.001), compared to the low resilience group (27.39 ± 7.434) as to the BAQ scores and among normal (14.48 ± 10.841, *p* = 0.035) and high resilience groups (17.09 ± 11.11, *p* = 0.011), compared to the low resilience group (10.93 ± 8.689) as to working experience. There were no statistically significant differences among the normal and high resilience groups (*p* = 0.862) as for the DAR-5 scores, the BAQ scores (*p* = 0.878), and working experience (*p* = 0.596). Levene’s test showed that the variances were equal (*p* > 0.05).

### 3.3. Two-Way Interaction between Psychological Resilience—Alcohol Abuse and Psychological Resilience—Gender in Terms of Aggression

**Figure 1:**
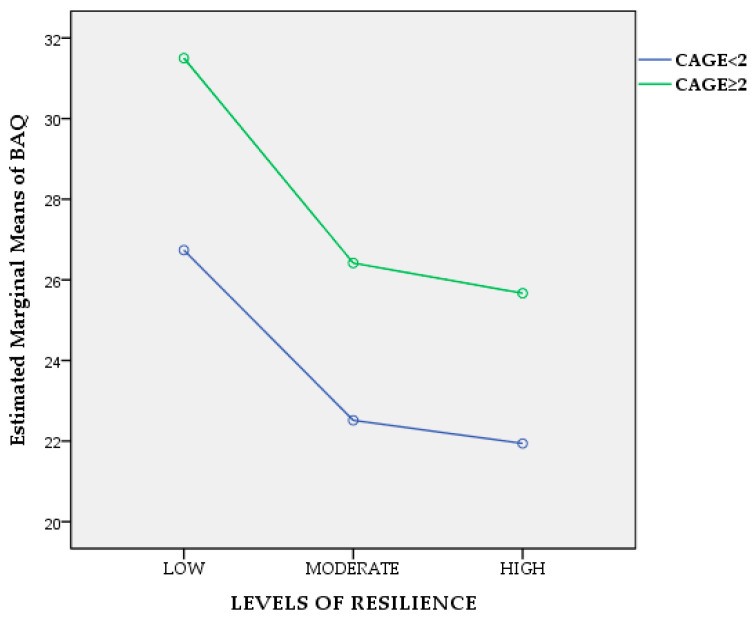
Line plot of alcohol abuse and levels of resilience for aggression.

**Figure 2:**
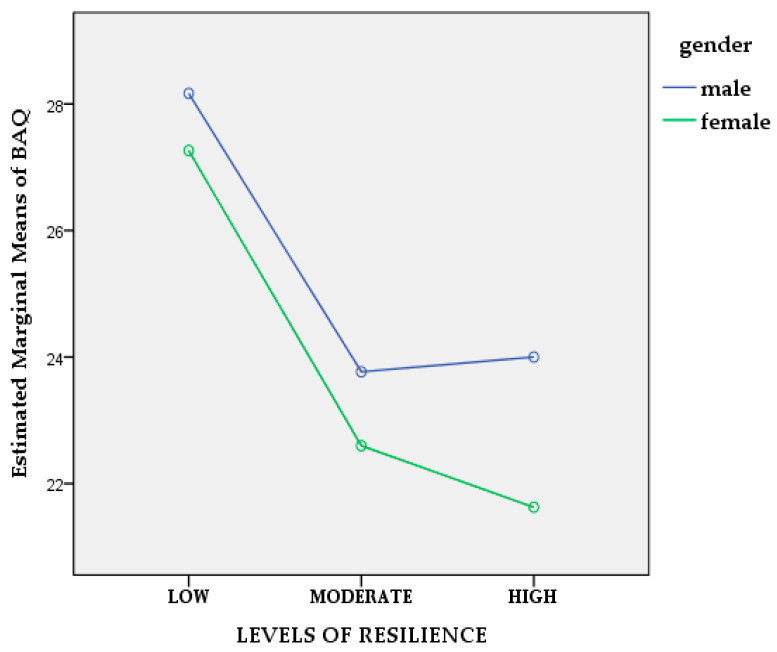
Line plot of gender and levels of resilience for aggression.

Since we are primarily interested in knowing if and how psychological resilience and alcohol abuse influence aggression, we employed a two-way ANCOVA to determine whether there is an interaction between psychological resilience and alcohol abuse in terms of aggression, after adjusting/controlling for age, working experience, and DAR-5 scores as covariates. Results reveal no statistically significant interaction between psychological resilience and alcohol abuse, whilst controlling for the above covariates, *F*_(2, 232)_ = 0.015, *p* = 0.985, partial η^2^ = 0.010, (Figure 1). Similarly, we investigated if gender interacts with psychological resilience in terms of aggression following the same procedure and results evidenced no statistically significant interaction between psychological resilience and gender, whilst controlling for age, working experience, DAR-5, and CAGE scores as covariates (*F*_(2, 231)_ = 0.891, *p* = 0.412, partial η^2^ =0.008), (Figure 2).

### 3.4. Correlations among Continuous Variables

Significant positive correlations were identified between DAR-5 and BAQ scores (*p* < 0.001) and negative between BRS scores with both DAR-5 and BAQ scores (*p* < 0.001). Age and work experience correlated positively with BRS scores (*p* < 0.001) and negatively with BAQ scores (*p* < 0.05). Scores on the CAGE questionnaire correlated positively with the BAQ scores (*p* < 0.05), (Table 3) and three out of the four BAQ subscales; physical aggression, verbal aggression, and anger (r = 0.187 **, *p* < 0.01; r = 0.142 *, *p* < 0.05; r = 0.139 *, *p* < 0.05, correspondingly). Scores on the hostility subscale of the BAQ were not related to CAGE scores (*p* > 0.05). Also, significant negative correlations were revealed among BRS and three out of the four BAQ subscales; hostility, verbal aggression, and anger (r = −0.435 **, *p* < 0.001; r= −0.170 **, *p* < 0.01; r= −0.292 **, *p* < 0.001, correspondingly). Scores on the physical aggression subscale of the BAQ were not related to BRS scores (*p* > 0.05). 

### 3.5. Multiple Regression Analysis

**Figure A1:**
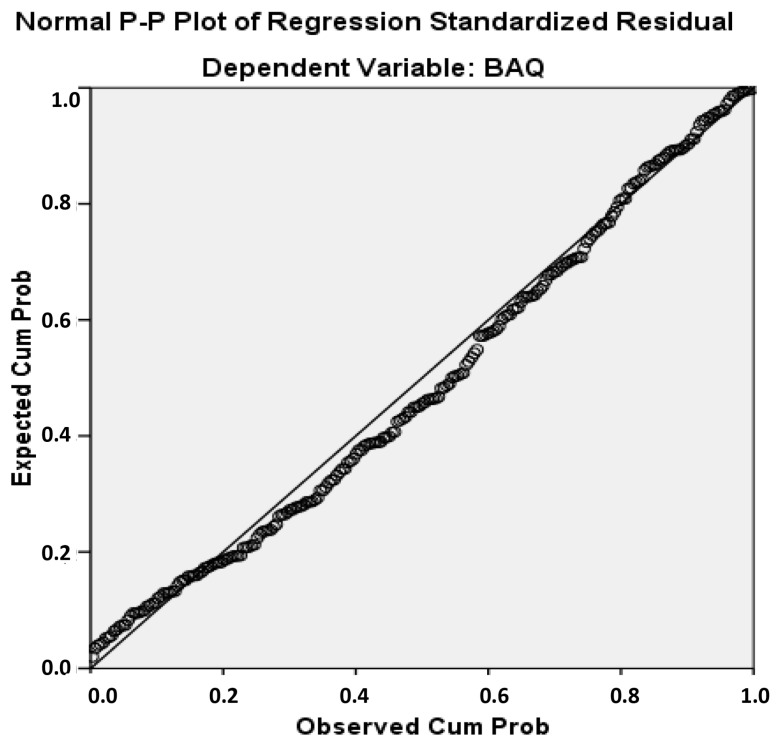
P-P plot to test the Normality of the distribution of the residuals.

**Figure A2:**
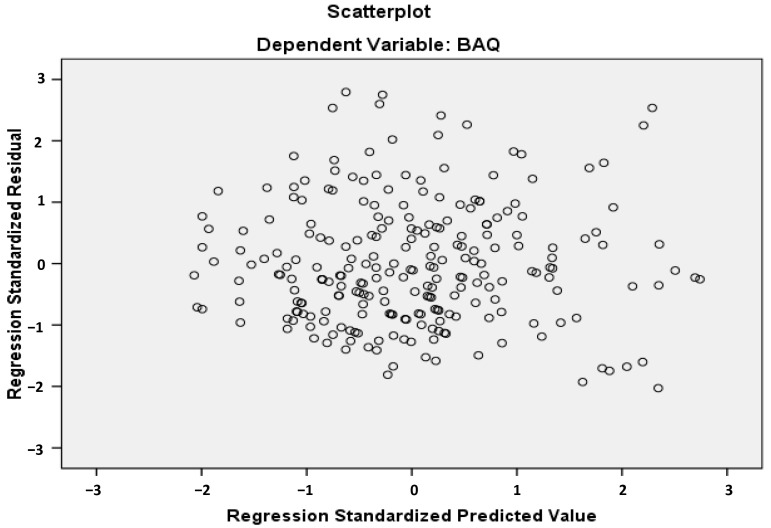
Scatterplot of the standardized residuals by the regression standardized predicted values to test homoscedasticity.

Stepwise multiple regression analysis was performed to evaluate the prediction of the BAQ scores (dependent variable) from the general characteristics of participants (age, gender, working experience, and profession), CAGE, DAR-5, and the BRS scores (independent variables). The assumptions of multiple regression analysis were satisfied. The independence of the residuals was checked with the Durbin–Watson test value being 2.291 (Table 4). The absence of multicollinearity in the data was checked with the Variance Inflation Factor (VIF), with values ranging from 1.013 to 1.150 (Table 4). Normality was checked by visual inspection of the predicted probability (P-P) plots to determine if the residuals of the regression (the errors between observed and predicted values) are normally distributed (depicted in Figure A1 which is included in Appendix A) and the scatterplots of residuals versus predicted values (plots of the standardized residuals by the regression standardized predicted value) to check for homoscedasticity (illustrated in Figure A2 which is also included in Appendix A, Breusch-Pagan test, *p* = 0.235). The regression analysis revealed that ‘scores on DAR-5′, ‘scores on BRS’, and ‘scores on CAGE’, gender and working experience were all significant predictors of ‘scores on the BAQ’, each explaining 16.4%, 5.9%, 2.1%, 1.7%, and 1.2% of the variance (Table 4). Participants’ predicted aggression was equal to 27.238 + 0.743 (DAR-5) − 1.939 (BRS) + 1.339 (CAGE) − 2.0003 (gender) − 0.074 (working experience).

### 3.6. Simple Mediation Analysis

**Figure 3:**
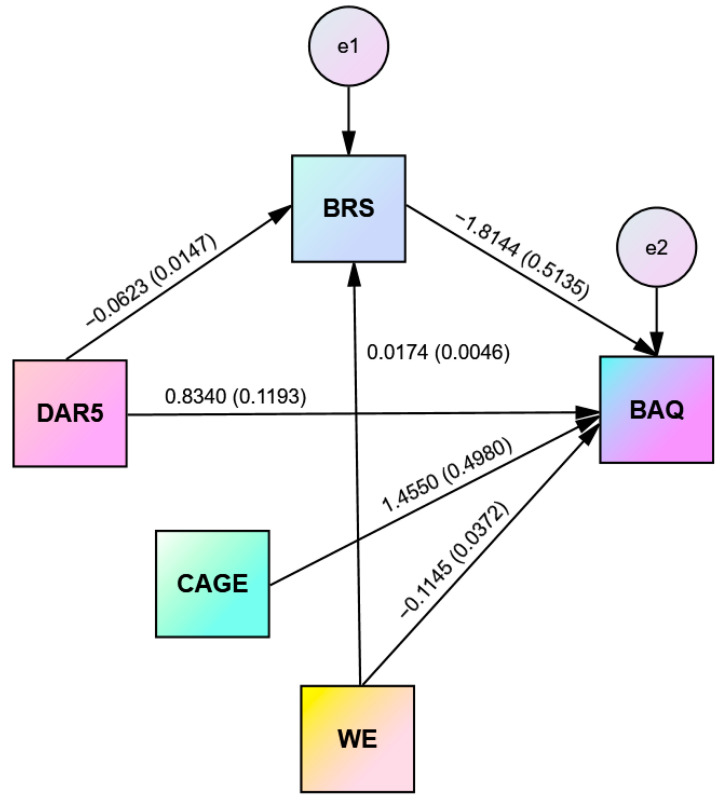
Mediation analysis of BRS on the DAR-5 and BAQ relationship with alcohol abuse and work experience (W.E.) as covariates.

In order to specify the nature of the relationship among significant variables identified from the regression analysis, we investigated the underlying mechanism by which anger influences aggression through psychological resilience in relation to alcohol abuse. A simple mediation analysis, using the bootstrap method, was conducted to test the hypothesis, setting BAQ as the dependent variable, DAR-5 as the predictor, and BRS as the mediator variable. Thus, the study assessed the mediating role of psychological resilience in the relationship between anger and aggression. Including covariates in the analysis is because we wanted to explain part of the variability in the outcome variable. The results revealed a significant indirect impact of anger on aggression through psychological resilience (b = 0.1130, t = 2.4145). Furthermore, the direct effect of anger on aggression in the presence of the mediator resilience was also found significant (b = 0.7211, *p* < 0.001). Hence, resilience partially mediated the relationship between anger and aggression and the model explains 13.55% of the variance in the outcome variable aggression (Table 5). Additionally, alcohol abuse proved to be a significant covariate affecting aggression but had an insignificant impact on resilience. Working experience, as a covariate, was found to have a significant impact on both aggression and resilience, but gender, in the presence of alcohol abuse and working experience as covariates, was found insignificant, and so in the last analysis, it was not included in the model. Unstandardized coefficients for the variables with standard errors in parentheses are illustrated in Figure 3. 

## 4. Discussion 

### 4.1. Summary of Results

During the first pandemic wave in this study one third of healthcare workers expressed high anger emotions, primarily prevalent in the female population and one out of ten respondents exhibited problematic alcohol use. Gender-specific types of aggression were identified with males displaying more physical aggression and females preferably reporting anger and hostility. Participants with current problematic alcohol use scored higher in physical and total aggression compared to those without alcohol use disorders. Psychological resilience presented negative associations with both anger and aggression and partially mediated the relationship between anger and aggression. Professional experience and alcohol abuse emerged as positive and negative risk factors contributing to aggression and psychological resilience.

Negative associations were identified between psychological resilience with the three of the aggression subscales, namely hostility, anger, and verbal aggression, which implies that increasing the level of resilience reduces the level of aggression. Among the three correlated with resilience dimensions of aggression, hostility had the strongest negative association with respect to the rest of the other subscales; results that are also supported by other research studies [101,102]. 

As expected, alcohol abuse presented positive associations with aggression, having the strongest correlation with physical aggression, but the only domain among the four aggressive domains that did not correlate with alcohol abuse was hostility, the cognitive domain of aggression. In our sample psychological resilience and alcohol abuse operate in different ways, primarily affecting distinct aggression domains, and no interaction was identified between these two constructs in terms of aggression. The fact that alcohol abuse impairs cognitive functions, specifically executive functions, information processing, and attentional control, among other individual differences, leading to alcohol-related aggression while psychological resilience requires integrity of self-regulation processes and intact prefrontal cortical control to achieve its salutary benefits, by taming aggressive urges, justifies the aforementioned results [50,103,104,105]. In other words, alcohol abuse undermines resilience’s protective role and fuels aggression, both directly and indirectly. 

### 4.2. Professional Experience and Demographic Variables as to Resilience and Workplace Aggression

Research suggests that work-related variables, like professional experience, is positively associated with resilience and studies evidence that the less experienced nurses have lower resilience compared with the higher experienced ones [59,106,107]. Work experiences can, under certain circumstances, empower employees, increasing job satisfaction, commitment, and performance and various studies confirm that over the years healthcare professionals may develop highly adaptive mechanisms, or resilient characteristics enabling them to consistently provide a high standard of care and remain competent despite unfavorable working conditions [108,109,110]. Other studies fail to provide evidence about the role of working experience as to resilience and support other implicated work-related and exogenous factors [111,112,113]. A recent systematic review revealed discrepancies while investigating factors associated with resilience and identified several positive related factors, like self-efficacy, social support and job satisfaction, and various negative such as stress, workplace bullying, and burnout [114]. 

Work experience, among other factors, predicted workplace aggression in healthcare workers attributed to the fact that the more experienced healthcare workers have eventually acquired skills enabling them to foresee, appraise, and resolve conflicts in a more competent way than their less experienced counterparts [115,116,117]. Previous research supported the increase in resilience with age since with advancing age life experiences are acquired [118]. Older people are deemed to have faced at times adverse life events that have enabled them to acquire a repertoire of effective coping skills [119]. Also, as to the rest of the demographic variables, results from most studies examining resilience in terms of gender are in favor of men, beyond reasons of heritability [120,121]. Gender-specific differences regarding resilience levels were not observed in our study. Furthermore, in other studies results were inconclusive and gender has been termed as an inconsistent and non-reliable predictor of resilience, possibly because measures of resilience are not gender sensitive [122].

### 4.3. Workplace Aggression and Interrelated Variables

Workplace aggression in healthcare is an immerging growing phenomenon as if the pandemic kindled an outrage against healthcare professionals. Previous research argued that being exposed to workplace aggression undoubtedly evokes emotional responses like anger, despair, or fear and the more harm-causing incidents of workplace aggression have a higher impact on female employees [123]. A review identified several staff-related causes of workplace aggression in healthcare: notably emotional dysregulation, distressing working conditions, unsupportive supervisors, inappropriate management, lack of instructions for handling aggressive incidents, and alcohol abuse [124]. In a recent qualitative study, many healthcare workers claimed that aggression in the workplace induced deleterious psychological and physical effects and reported experiencing fatigue, exhaustion, and distress effectuating a negative influence in providing effective and safe patient care [125]. 

### 4.4. Alcohol Abuse among Healthcare Professionals

Another alarming trend is problematic alcohol use among physicians, which appears to have been increasing during the pandemic [126,127]. A recent systematic literature review revealed a high rate of self-reported alcohol use disorder among doctors impacting up to one third of them [128]. According to this review, the rate of reported alcohol use disorder increased from 16.3% between 2006 and 2010 to 26.8% between 2017 and 2020. The proportion of a positive screen using the CAGE questionnaire was 3.8% to 22.0% for studies [128]. In a cross-sectional study from a hospital during the first wave of the pandemic, 17.1% of healthcare professionals admitted increased alcohol consumption assumed to be a maladaptive coping strategy from vulnerable individuals who lack adequate work experience while attempting to handle occupational stress and anxiety [129]. Increased alcohol consumption has also been reported among nursing staff from diverse healthcare settings mostly due to increased workplace stress [130].

### 4.5. The Role of Psychological Resilience as to Aggression and Alcohol Abuse 

It has been argued that the pandemic created the optimal setting for testing psychological resilience in the presence of worldwide challenges heavily impacting mental health. A recent review examining resilience among frontline healthcare workers during the pandemic evidenced modest resilience scores [131]. According to a meta-analysis the pandemic adversely affected the psychological well-being of healthcare workers worldwide and among other disappointing results, they reported that the rate of low resilience was 16.1% (95% CI: 12.8–19.4) [132]. It is generally accepted that resilience is amenable to change and can be further enhanced by interventions, such as cognitive–behavioral, stress inoculation, acceptance and commitment, mindfulness, and problem-solving psychotherapies [133,134].

Resilience has an important role in the resolution of psychological difficulties such as aggression, depression, or anxiety, caused by early adversities and allows people to deal with emotional and behavioral reactions and, therefore, reduces the likelihood for displaying aggressive behaviors when confronted with any distressing situation [135]. Levels of resilience and aggression are greatly influenced by diverse social, biological, environmental, situational, personal, and cultural factors. Protective and risk factors that modulate patterns of response to lifetime experiences and determine the correlation between resilience and aggression are identified, such as self-control, gender, coping styles, family and social relationships, and socioeconomic status, among others [136]. 

Studies highlight that people who are inclined to risk-taking behaviors, such as alcohol and substance use, aggressive, and other disruptive behaviors, have maladaptive coping styles and are not capable of managing life stressors with resilience [137]. In other words, low resilience levels may lead to inefficient management, as in the case of excessive alcohol consumption to overcome a variety of stressors [138,139]. A recent cross-sectional survey identified resilience as a moderator, compensating for the increase in alcohol use arising from the pandemic-related stress [140]. 

Likewise, investigating the association between resilience and various psychosocial factors, it was reported that resilience has a negative correlation with anger expression and was also implicated in emotion regulation and impulse control [141,142]. This sense of emotional regulation allows a resilient person to withstand during adversities instead of reacting aggressively [143]. Resilience also encourages people to seek healthier ways to relieve from stress, using relaxation and exercising, and ultimately this contributes to the reduction of negative emotions [144]. Furthermore, a research study supported that enhancing resilience in the workplace is also of great importance, as improving the level of resilience increases the probability of diminishing undesirable workplace behaviors, such as anger, hostility towards co-workers, or verbal aggression, ultimately effectuating better employee performance [145].

### 4.6. Anger and Aggression as to Gender

In our sample, male respondents demonstrated higher levels of self-reported physical aggression compared with females and female participants exhibited higher scores in self-reported levels of anger and hostility, in agreement with other research [146]. There is consensus in the literature that anxiety symptoms tend to be associated with increased levels of anger and hostility [147,148], although most people with anxiety problems are less likely to express themselves with overt aggression due to the fear of negative evaluation by others. Sometimes people in their work environment are potentially confronted with morally injurious experiences, which may challenge fundamental ethical principles, placing them at increased risk of presenting symptoms of anxiety and behavioral problems such as anger and hostility [149,150]. In this sense, anger is a frequent reaction to potentially fatal situations encountered at work, and results from studies during the pandemic suggest that healthcare workers experience increased feelings of anger as a result of direct or indirect contact with COVID-19 patients and proximity of exposure to these patients and the female gender were identified as risk factors [151]. Similar conclusions emerge from previous research that focused on other life-threatening situations experienced by female military medical personnel [152]. 

Literature suggests that there are differences in aggression according to gender, with men displaying direct and women indirect aggression [153]. Biological, psychological, and social factors have been associated with these gender-specific differences [154]. From an evolutionary perspective, women tend to be more fearful and risk-averse than men, with a greater vulnerability to developing anxiety disorders [49]. These gender differences in fear partly explain differences in physical aggression. Although women may be just as likely to experience anger as men, they are assumed to use non-injurious ways of expressing anger and also express different beliefs about injurious modes of aggression [155]. Women view direct aggression as a failure of self-control and men as a means of controlling others [156]. Also, research results support that in intoxicated states alcohol increases aggression mostly in men and in individuals predisposed to aggressive behavior [157]. In general, participants predisposed to aggression tend to perceive conflict and attempt to exercise inhibitory control. Research suggests that females with high trait hostility exhibit a pattern of increased inhibitory control, whereas men with high hostility do not display this response, pointing to gender differences in aggressive behavior [158]. Another study revealed impaired frontal interhemispheric connectivity in women in response to alcohol consumption, suggesting a plausible explanation for aggression in the female gender [159]. 

Upon provocation, the gender effect on aggression may be blunted. Angry rumination triggered by interpersonal provocation reduces self-control and raises aggression [160]. Angry rumination, one of the important aggression-related cognitive factors, perpetuates anger and increases aggression by reducing self-control and research studies suggest that anger rumination mediates the effect of trait hostility and trait anger on aggression [161,162,163]. 

### 4.7. Psychotherapeutic Interventions Targeting Anger, Aggression, Hostility and Substance Abuse

Through diverse proposed mechanisms, mindfulness may counteract angry rumination and decrease aggression, anger, and hostility [164]. Left untreated, according to a recent meta-analysis of longitudinal studies, hostility and anger elevate the risk of coronary artery disease among healthy subjects and predict poorer prognosis among cardiac patients [165]. Also, other research supported that the cognitive dimensions of hostility and anger may anticipate and predispose to depressive symptoms [166]. 

Another example of psychotherapeutic interventions, from a cognitive–behavioral approach, suggests that stress-related mental disorders like depression, anxiety disorder, and substance abuse, can be viewed as the outcome of dysfunctional thinking patterns. When people are exposed to stressful situations, they often exhibit maladaptive behavioral reactions and suffer from negative emotions because of dysfunctional cognitions. This is consistent with other theories of stress and resilience, suggesting that it is the way the stressor is perceived that drives stress reactions. Consequently, modifying cognitive processes to more adaptive thought patterns is likely to adjust both behavioral and emotional responses to stress. Questioning maladaptive thinking patterns and introducing novel strategies eventually promotes the resilience factors of cognitive flexibility and adaptive coping [167]. 

Cognitive–behavioral models of addiction and relapse treatment emphasize the role of adverse emotional experiences as determinants of alcohol use and relapse, and research suggests that being able to communicate negative emotional states, a feature of psychological resilience, prevents alcohol use disorders [168]. Resilience building interventions against stress, promoting self-awareness, enabling cognitive restructuring, and encouraging interpersonal relations could advance effective stress management and help individuals avoid alcohol use problems or prevent relapse [169].

### 4.8. Recommendations

Primary, secondary, and tertiary are the three levels of addressing healthcare workers’ resilience; with primary interventions targeting coping strategies and facilitating communication skills, while secondary interventions screening for burnout and taking care of those at risk should be implemented [170]. Finally, tertiary-level interventions should reach out to healthcare workers who operate beyond safety limits and require treatment in order to recover [170]. The National Academies of Sciences, Engineering, and Medicine Initiative for Clinician Wellbeing and Resilience recommends interventions both at the individual and the organizational level. At the individual level, interventions include good sleep hygiene, physical exercise programs, social support, mindfulness practice and stress management approaches, reflective counseling, etc. [171]. Interventions at the organizational level involve education training sessions, enabling information dissemination regarding the principles of psychosocial resilience, establishing contact between psychosocial services and health professionals, and ensuring the feedback of their worries and proposals [171]. At present, there is limited research evidence to offer recommendations to promote the resilience of health care workers [172]. Conducting robust research is essential to identify optimal approaches for building resilience among healthcare workers and to further explore how healthcare settings should be integrated into interventions [173].

Another critical issue to attend to is workplace aggression. Occupational protocols and administrative strategies are needed that target safety issues, monitoring, and referral of events, as well as workforce training on the prevention and management of workplace aggression [115]. It is strongly advised to enhance communication skills, stress relief, and prevent or manage confrontation through educational training programs for healthcare workers and to provide explicit guidelines for the referral of aggressive incidents in the workplace [174,175,176,177,178]. Preventive interventions to reduce workplace aggression should be evaluated at regular intervals to provide feedback on the benefits and drawbacks of implemented strategies [179]. Also, it is suggested that aggression management be incorporated into the undergraduate nursing education programs and effective strategies be integrated to enhance and improve nursing students’ resilience when dealing with aggressive incidents in the clinical setting [180].

Providing healthcare workers with appropriate training to improve self-control in stressful situations and upgrade their social competencies will diminish aggressive incidents, while enabling feedback and support from their supervisors will increase their sense of security and boost their self-confidence [108,181]. Measures to address workplace aggression should also entail strategies to mitigate the negative impact of verbal and physical aggression in the workplace on healthcare workers [182]. Further, in order to create a safe working environment, it is essential to have good quality and efficient security staff to reduce exposure to potential aggressors [183]. Health authorities and policymakers need to implement strategies focusing on specifically identified factors within the psychosocial framework, considering both proximal and distal risk factors, both patient and healthcare-related determinants of workplace aggression [37]. To draw conclusions about the effect of these specific approaches or programs on reducing workplace aggression, interventional studies that evaluate the proposed initiatives are needed [184]. 

For alcohol use disorders among healthcare workers, it is important that both diagnostic tests and therapeutic interventions are implemented as an appropriate approach to this important health problem [185]. These programs targeting high-risk populations should include monitoring, screening, and counseling on the harmful effects of alcohol consumption, and health education about healthy nutrition, sleep quality, physical exercise, and stress reduction techniques to dissuade alcohol use as a management strategy [4,186]. Health education training and counseling interventions should be personalized and adjusted to the population at risk [187]. Longitudinal population studies employing national health data are required to allow relevant comparisons in order to identify high-risk groups for harmful alcohol use and provide appropriate support [188,189]. 

Finally, the workplace should be monitored for stress problems on an ongoing basis, in order to early detect and address these problems, but also to promote a healthy work environment and reduce the harmful aspects of work [190]. The resilience of health systems necessitates drastic structural and resource reforms, along with facilitating health workers’ access to psychological support. The context-specific design of intervention programs should be based on informed inputs and observational outcomes from research studies aimed at uncovering the pressures faced by health care workers and their mental health-related demands [191,192]. Drafting psychological support programs and interventions plans calls for collaboration with health professionals in order to identify work stressors and determine support needs [193]. Knowing the areas of distress that affect human resources in healthcare settings is particularly critical to ensuring the resilience of health systems [173]. 

### 4.9. Limitations

In our study, we examined the role of psychological resilience on anger and aggression in relation to alcohol abuse among healthcare professionals, but apart from psychological resilience, there are other identified protective factors such as family and social support, along with negative risk factors such as anxiety, depression, and burnout prevalent among healthcare professionals, particularly among those allocated on the front line of the pandemic. To draw effective individualized psychological interventions all these factors, not taken into account in our study, should be considered. Other limitations in the present study include the limited sample size which impedes the drawing of safe epidemiological conclusions; the convenience sampling method possibly reflecting selection bias; the gender disproportionality of participants, which may have affected the generalizability of the results; employing self-administered questionnaires which raises concerns about the objectivity of the estimates; the cross-sectional design of the study which precludes causal inferences and online data collection excluding healthcare professionals without internet access and further influencing the generalizability of the results. 

## 5. Conclusions

The present study confirmed the protective role of psychological resilience neutralizing anger experiences and averting aggressive tendencies among healthcare professionals during the first pandemic wave. Further, professional experience and alcohol abuse were identified as positive and negative risk factors, correspondingly, impacting aggression and psychological resilience and explaining part of the variability in aggression. Implementing interventions to address workplace aggression and alcohol use disorders among healthcare workers and enhance resilience to counteract the harmful aspects of work is of outmost importance. 

## Figures and Tables

**Table 1:** General characteristics of participants and DAR-5, BRS, CAGE, BAQ, and subscales scores as to gender.

Participants	Men N = 53		Women N = 190		Total N = 243	
Descriptive Statistics	Mean	SD	Mean	SD	Mean	SD
Age	42.11	9.89	41.49	9.38	41.63	9.48
Working experience (in years)	13.23	11.04	15.54	10.58	15.03	10.7
Dimensions of Anger Reactions-5 (DAR-5)	9.38 *	2.52	10.37 *	3.49	10.16	3.33
Brief Resilience Scale (BRS)	3.79	0.72	3.56	0.81	3.61	0.8
CAGE questionnaire	0.36	0.68	0.41	0.83	0.4	0.798
Brief Aggression Questionnaire (BAQ)	24.34	5.56	23.33	7.26	23.55	6.93
Physical Aggression (PA)	5.45 **	2.47	4.11 **	1.87	4.4	2.09
Verbal Aggression (VA)	7.04	2.44	6.54	2.62	6.65	2.6
Hostility (H)	5.72 **	2.14	6.72 **	2.61	6.5	2.54
Anger (A)	6.13	2.22	5.96	2.56	6.0	2.49

NOTE: * *p* < 0.05; ** *p* < 0.01.

**Table 2:** General characteristics of participants and DAR-5, BRS, BAQ, and subscales scores as to alcohol abuse.

Participants	CAGE < 2 N = 219		CAGE ≥ 2 N = 24	
Descriptive Statistics	Mean	SD	Mean	SD
Age	41.6	9.5	41.88	9.53
Working experience (in years)	13.88	10.7	16.38	10.95
Dimensions of Anger Reactions-5 (DAR-5)	10.09	3.37	10.75	2.95
Brief Resilience Scale (BRS)	3.63	0.77	3.44	1.008
Brief Aggression Questionnaire (BAQ)	23.11 *	6.7	27.5 *	7.95
Physical Aggression (PA)	4.24 **	1.95	5.83 **	2.745
Verbal Aggression (VA)	6.55	2.54	7.5	2.87
Hostility (H)	6.42	2.54	7.21	2.52
Anger (A)	5.89	2.38	6.96	3.237

NOTE: * *p* < 0.05; ** *p* < 0.01.

**Table 3:** Correlations among age, work experience, BAQ, DAR-5, BRS, and CAGE.

Pearson Correlation Participants = 243		AGE	W.E.	CAGE	BAQ	DAR-5
Working Experience (W.E.)	r	0.861 **				
	*p*	0.000				
CAGE	r	0.037	0.058			
	*p*	0.561	0.374			
Brief Aggression Questionnaire (BAQ)	r	−0.144 *	−0.144 *	0.186 **		
	*p*	0.025	0.025	0.004		
Dimensions of Anger Reactions-5 (DAR-5)	r	−0.002	0.058	0.075	0.403 **	
	*p*	0.981	0.367	0.247	0.000	
Brief Resilience Scale (BRS)	r	0.207 **	0.213 **	−0.073	−0.333 **	−0.251 **
	*p*	0.001	0.001	0.260	0.000	0.000

NOTE: * *p* < 0.05; ** *p* < 0.01.

**Table 4:** Stepwise multiple regression (only statistically significant variables are included).

Dependent Variable: Brief Aggression Questionnaire	B	R Square	R Square Change	Beta	*t*	*p*	VIF	Durbi-Watson
(Constant)	27.238				9.127	0.000 **		2.291
Dimensions of Anger Reactions-5	0.743	0.164	0.164	0.359	6.172	0.000 **	1.092	
Brief Resilience Scale	−1.939	0.223	0.059	−0.225	−3.778	0.000 **	1.150	
CAGE	1.339	0.244	0.021	0.155	2.767	0.006 **	1.013	
Gender	−2.003	0.261	0.017	−0.120	−2.123	0.035 *	1.036	
Working Experience	−0.074	0.273	0.012	−0.115	−1.993	0.047 *	1.080	

Notes: B = unstandardized coefficients; Beta = standardized regression coefficient; correlations are statistically significant at the * *p* < 0.05 or ** *p* < 0.01 level.

**Table 5:** Mediation Analysis of BRS on the DAR-5 and BAQ relationship with Alcohol Abuse and Work Experience (W.E.) as Covariates.

Variable	b	SE	t	*p*	95% Confidence Interval	
					LLCI	ULCI
DAR-5 -> BRS	−0.0623	0.0147	−4.2248	0.0000	3.6811	4.3446
DAR-5 -> BAQ	0.8340	0.1193	6.9913	0.0000	0.5990	1.0691
DAR-5 -> BRS -> BAQ	−1.8144	0.5135	−3.5332	0.0005	0.4831	0.9591
Covariates						
CAGE-> BAQ	1.4550	0.4980	−2.9218	0.0038	0.4740	2.4361
W.E. -> BRS	0.0174	0.0046	3.7902	0.0084	0.1776	0.0265
W.E. -> BAQ	−0.1145	0.0372	−3.0807	0.0023	−0.1878	−0.0413
Effects						
Direct	0.7211	0.1208	5.9684	0.0000	0.4831	0.9591
Indirect *	0.1130	0.0486	2.4145		0.0382	0.2213
Total	0.8340	0.1193	6.9913	0.0000	0.5990	1.0691

* Based on 5000 bootstrap samples. Abbreviations: W.E., working experience (in years).

## Data Availability

The data that support the findings of this study are available from the corresponding author, [A.T.], upon reasonable request.
